# Supplementary material for: End Sequence Analysis Toolkit (ESAT) expands the extractable information from single-cell RNA-seq data
Source: Genome Res. 2016 Oct;26(10):1397–410. doi: 10.1101/gr.207902.116 (PMC5052061; doi:10.1101/gr.207902.116)
Supplement: Supplemental Material [file supp_gr.207902.116_Supp_Materials.tar.gz › ESAT-esat_v0.1/doc/html/classumms_1_1core_1_1model_1_1score_1_1_sum_score.html]

ESAT: umms.core.model.score.SumScore Class Reference


ESAT


- Main Page
- Related Pages
- Packages
- Classes
- Files

- Class List
- Class Index
- Class Hierarchy
- Class Members

All Classes Namespaces Files Functions Variables Enumerator Pages

- umms
- core
- model
- score
- SumScore

Classes |
Public Member Functions |
List of all members

umms.core.model.score.SumScore Class Reference

Inheritance diagram for umms.core.model.score.SumScore:

[legend]

Collaboration diagram for umms.core.model.score.SumScore:

[legend]

|  |  |
| --- | --- |
| Classes | |
| class | **Processor** |
|  | |

|  |  |
| --- | --- |
| Public Member Functions | |
|  | SumScore (Annotation a) |
|  | |
|  | SumScore (AnnotationCollection<?extends Annotation > model, Annotation region) |
|  | |
| double | getScore () |
|  | |
| Public Member Functions inherited from umms.core.model.score.AbstractListScore | |
|  | AbstractListScore (Annotation a) |
|  | |
| void | setScores (List< Double > scores) |
|  | |
| double | getScore () |
|  | |
| double | getCount () |
|  | |
| double | getSum () |
|  | |
| double | getMedian () |
|  | |
| double | getMean () |
|  | |
| double | getStandardDeviation () |
|  | |
| double[] | getScores () |
|  | |
| String | toString () |
|  | |
| Public Member Functions inherited from umms.core.model.score.WindowScore.AbstractWindowScore | |
|  | AbstractWindowScore (Annotation t) |
|  | |
| Annotation | getAnnotation () |
|  | |

|  |  |
| --- | --- |
| Additional Inherited Members | |
| Static Public Attributes inherited from umms.core.model.score.AbstractListScore | |
| static double | DEFAULT\_VALUE = -1.0 |
|  | |
| Protected Attributes inherited from umms.core.model.score.WindowScore.AbstractWindowScore | |
| Annotation | annotation |
|  | |

## Detailed Description

Author
:   engreitz This class calculates statistics (sum, average, stddev, median, etc.) on the scores of annotations in the given region, in addition to counting the number of annotations.

## Constructor & Destructor Documentation

|  |  |  |  |  |  |
| --- | --- | --- | --- | --- | --- |
| umms.core.model.score.SumScore.SumScore | ( | Annotation | *a* | ) |  |

|  |  |  |  |
| --- | --- | --- | --- |
| umms.core.model.score.SumScore.SumScore | ( | AnnotationCollection<?extends Annotation > | *model*, |
|  |  | Annotation | *region* |
|  | ) |  |  |

Here is the call graph for this function:

## Member Function Documentation

|  |  |  |  |  |
| --- | --- | --- | --- | --- |
| double umms.core.model.score.SumScore.getScore | ( |  | ) |  |

Returns the number of reads overlapping this region

Returns
:   number of reds overlapping this window Returns the "score" as defined by each subclass (e.g. count, ratio, sum of annotation scores, etc.)

Implements umms.core.model.score.WindowScore.

Here is the call graph for this function:

---

The documentation for this class was generated from the following file:

- java/umms/core/model/score/SumScore.java


---

Generated on Fri Aug 15 2014 15:11:14 for ESAT by  

 1.8.7
